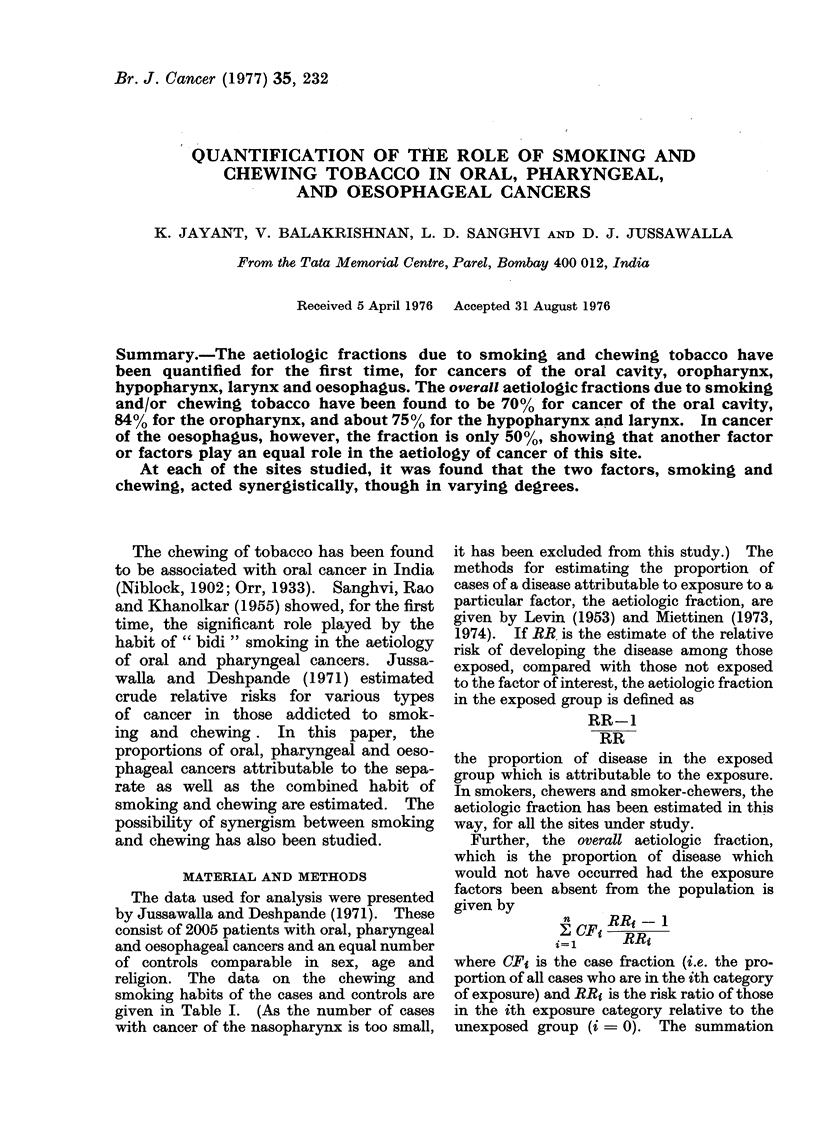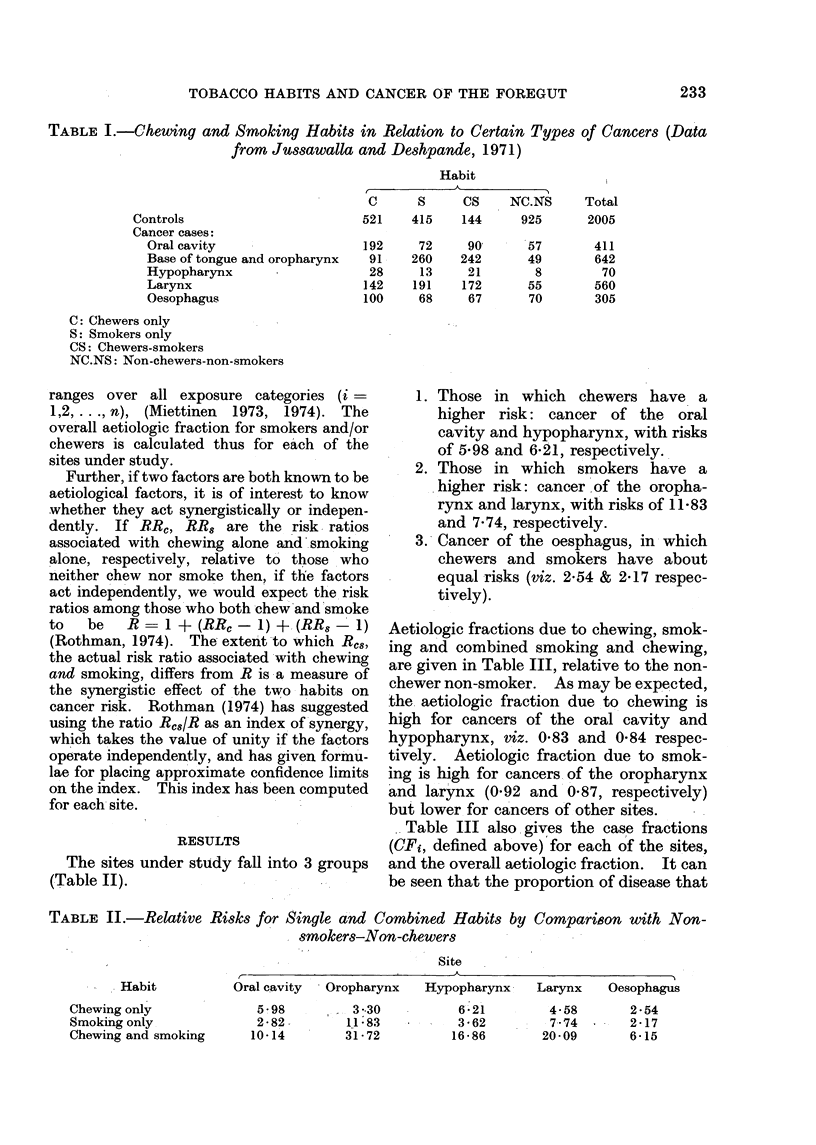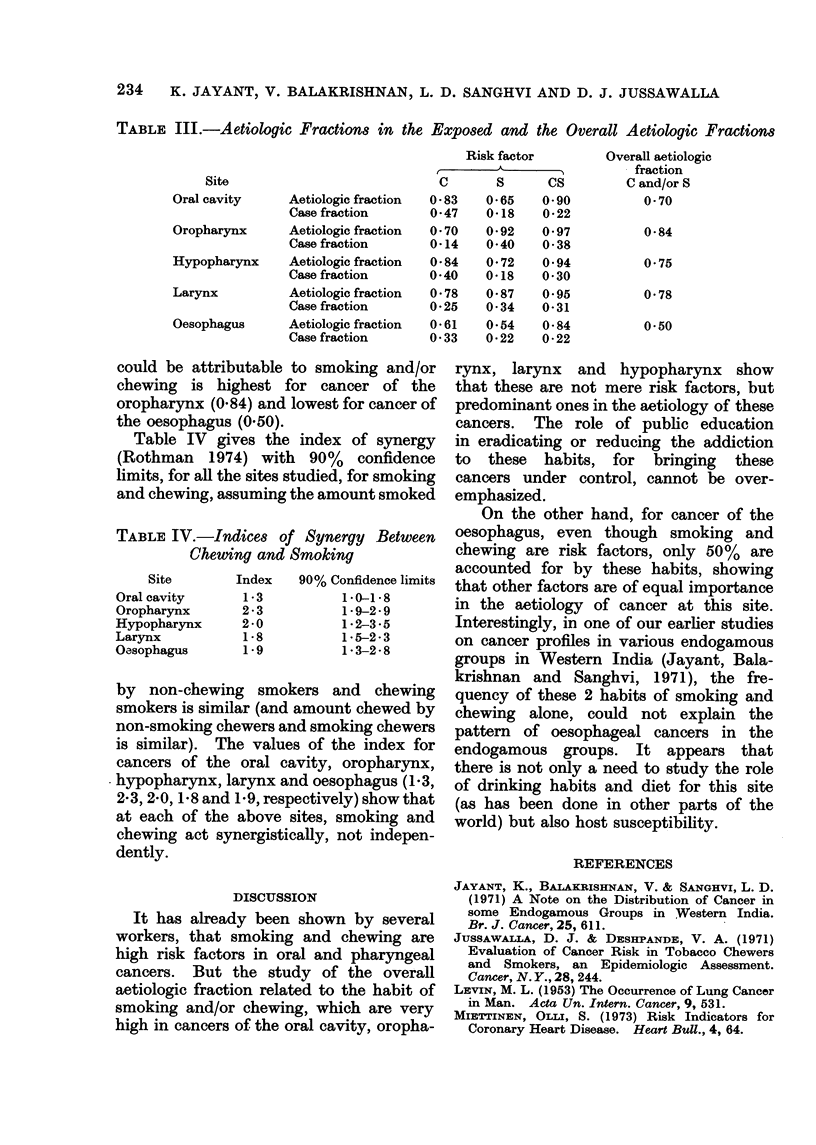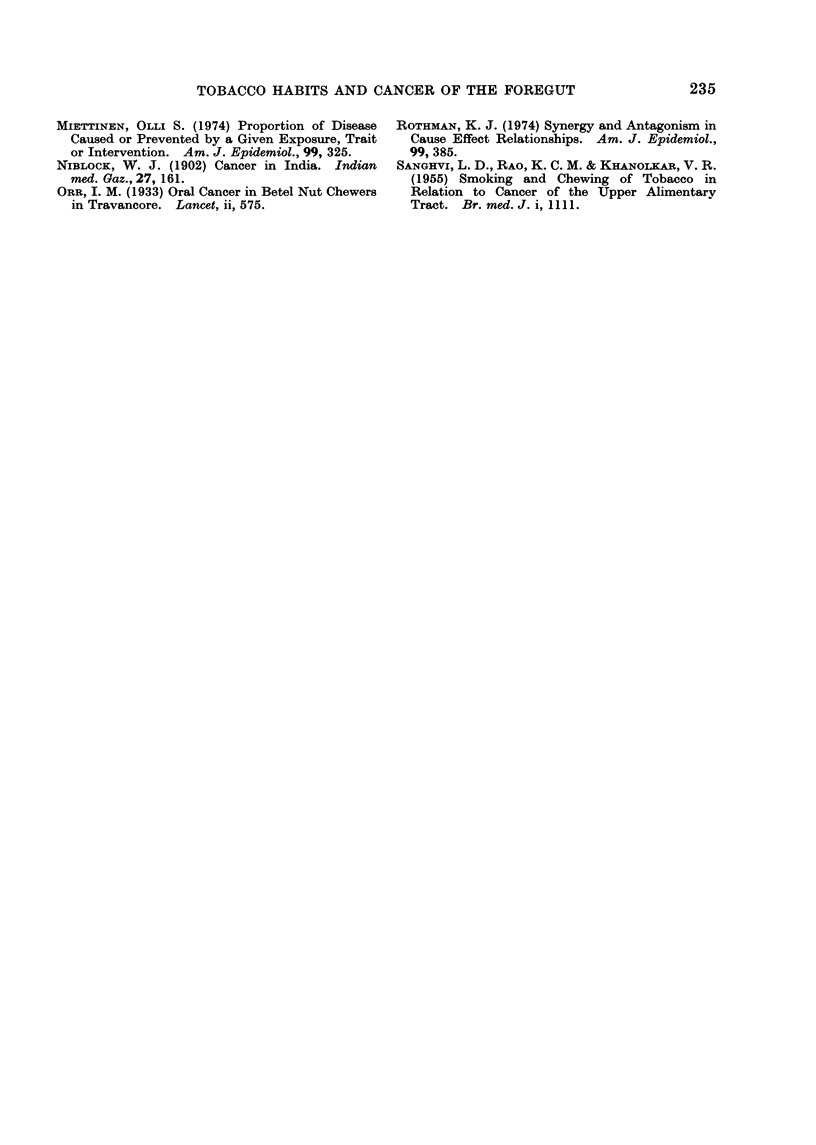# Quantification of the role of smoking and chewing tobacco in oral, pharyngeal, and oesophageal cancers.

**DOI:** 10.1038/bjc.1977.31

**Published:** 1977-02

**Authors:** K. Jayant, V. Balakrishnan, L. D. Sanghvi, D. J. Jussawalla

## Abstract

The aetiologic fractions due to smoking and chewing tobacco have been quantified for the first time, for cancers of the oral cavity, oropharynx, hypopharynx, larynx and oesophagus. The overall aetiologic fractions due to smoking and/or chewing tobacco have been found to be 70% for cancer of the oral cavity, 84% for the oropharynx, and about 75% for the hypopharynx and larynx. In cancer of the oesophagus, however, the fraction is only 50%, showing that another factor or factors play an equal role in the aetiology of cancer of this site. At each of the sites studied, it was found that the two factors, smoking and chewing, acted synergistically, though in varying degrees.


Br. J. Cancer (1977) 35, 232

### QUANTIFICATION OF THE ROLE OF SMOKING AND CHEWING TOBACCO IN ORAL, PHARYNGEAL, AND OESOPHAGEAL CANCERS

K. JAYANT, V. BALAKRISHNAN, L. D. SANGHVI AND D. J. JUSSAWALLA

*From the Tata Memorial Centre, Parel, Bombay* 400 012, *India*

Received 5 April 1976 Accepted 31 August 1976

**Summary.—The aetiologic fractions due to smoking and chewing tobacco have been quantified for the first time, for cancers of the oral cavity, oropharynx, hypopharynx, larynx and oesophagus. The *overall* aetiologic fractions due to smoking and/or chewing tobacco have been found to be 70% for cancer of the oral cavity, 84% for the oropharynx, and about 75% for the hypopharynx and larynx. In cancer of the oesophagus, however, the fraction is only 50%, showing that another factor or factors play an equal role in the aetiology of cancer of this site.**

**At each of the sites studied, it was found that the two factors, smoking and chewing, acted synergistically, though in varying degrees.**

The chewing of tobacco has been found to be associated with oral cancer in India (Niblock, 1902; Orr, 1933). Sanghvi, Rao and Khanolkar (1955) showed, for the first time, the significant role played by the habit of " bidi " smoking in the aetiology of oral and pharyngeal cancers. Jussawalla and Deshpande (1971) estimated crude relative risks for various types of cancer in those addicted to smoking and chewing. In this paper, the proportions of oral, pharyngeal and oesophageal cancers attributable to the separate as well as the combined habit of smoking and chewing are estimated. The possibility of synergism between smoking and chewing has also been studied.

### MATERIAL AND METHODS

The data used for analysis were presented by Jussawalla and Deshpande (1971). These consist of 2005 patients with oral, pharyngeal and oesophageal cancers and an equal number of controls comparable in sex, age and religion. The data on the chewing and smoking habits of the cases and controls are given in Table I. (As the number of cases with cancer of the nasopharynx is too small, it has been excluded from this study.) The methods for estimating the proportion of cases of a disease attributable to exposure to a particular factor, the aetiologic fraction, are given by Levin (1953) and Miettinen (1973, 1974). If *RR* is the estimate of the relative risk of developing the disease among those exposed, compared with those not exposed to the factor of interest, the aetiologic fraction in the exposed group is defined as

$$\frac{\mathrm{RR}-1}{\mathrm{RR}}$$

the proportion of disease in the exposed group which is attributable to the exposure. In smokers, chewers and smoker-chewers, the aetiologic fraction has been estimated in this way, for all the sites under study.

Further, the *overall* aetiologic fraction, which is the proportion of disease which would not have occurred had the exposure factors been absent from the population is given by

$$\sum_{i=1}^{n} CF_i \frac{RR_i - 1}{RR_i}$$

where $CF_i$ is the case fraction (*i.e.* the proportion of all cases who are in the *i*th category of exposure) and $RR_i$ is the risk ratio of those in the *i*th exposure category relative to the unexposed group ($i = 0$). The summation

TABLE I.—*Chewing and Smoking Habits in Relation to Certain Types of Cancers (Data from Jussawalla and Deshpande,* 1971)

| | Habit | | | | |
|---|---|---|---|---|---|
| | C | S | CS | NC.NS | Total |
| Controls | 521 | 415 | 144 | 925 | 2005 |
| Cancer cases: | | | | | |
| Oral cavity | 192 | 72 | 90 | 57 | 411 |
| Base of tongue and oropharynx | 91 | 260 | 242 | 49 | 642 |
| Hypopharynx | 28 | 13 | 21 | 8 | 70 |
| Larynx | 142 | 191 | 172 | 55 | 560 |
| Oesophagus | 100 | 68 | 67 | 70 | 305 |

C: Chewers only
S: Smokers only
CS: Chewers-smokers
NC.NS: Non-chewers-non-smokers ranges over all exposure categories ($i = 1,2,\ldots,n$), (Miettinen 1973, 1974). The overall aetiologic fraction for smokers and/or chewers is calculated thus for each of the sites under study.

Further, if two factors are both known to be aetiological factors, it is of interest to know whether they act synergistically or independently. If $RR_c$, $RR_s$ are the risk ratios associated with chewing alone and smoking alone, respectively, relative to those who neither chew nor smoke then, if the factors act independently, we would expect the risk ratios among those who both chew and smoke to be $R = 1 + (RR_c - 1) + (RR_s - 1)$ (Rothman, 1974). The extent to which $R_{cs}$, the actual risk ratio associated with chewing *and* smoking, differs from $R$ is a measure of the synergistic effect of the two habits on cancer risk. Rothman (1974) has suggested using the ratio $R_{cs}/R$ as an index of synergy, which takes the value of unity if the factors operate independently, and has given formulae for placing approximate confidence limits on the index. This index has been computed for each site.

### RESULTS

The sites under study fall into 3 groups (Table II).

1. Those in which chewers have a higher risk: cancer of the oral cavity and hypopharynx, with risks of 5·98 and 6·21, respectively.
2. Those in which smokers have a higher risk: cancer of the oropharynx and larynx, with risks of 11·83 and 7·74, respectively.
3. Cancer of the oesphagus, in which chewers and smokers have about equal risks (*viz.* 2·54 & 2·17 respectively).

Aetiologic fractions due to chewing, smoking and combined smoking and chewing, are given in Table III, relative to the non-chewer non-smoker. As may be expected, the aetiologic fraction due to chewing is high for cancers of the oral cavity and hypopharynx, *viz.* 0·83 and 0·84 respectively. Aetiologic fraction due to smoking is high for cancers of the oropharynx and larynx (0·92 and 0·87, respectively) but lower for cancers of other sites.

Table III also gives the case fractions ($CF_i$, defined above) for each of the sites, and the overall aetiologic fraction. It can be seen that the proportion of disease that

TABLE II.—*Relative Risks for Single and Combined Habits by Comparison with Non-smokers–Non-chewers*

| | Site | | | | |
|---|---|---|---|---|---|
| Habit | Oral cavity | Oropharynx | Hypopharynx | Larynx | Oesophagus |
| Chewing only | 5·98 | 3·30 | 6·21 | 4·58 | 2·54 |
| Smoking only | 2·82 | 11·83 | 3·62 | 7·74 | 2·17 |
| Chewing and smoking | 10·14 | 31·72 | 16·86 | 20·09 | 6·15 |

TABLE III.—*Aetiologic Fractions in the Exposed and the Overall Aetiologic Fractions*

| Site | | Risk factor C | S | CS | Overall aetiologic fraction C and/or S |
|---|---|---|---|---|---|
| Oral cavity | Aetiologic fraction | 0·83 | 0·65 | 0·90 | 0·70 |
| | Case fraction | 0·47 | 0·18 | 0·22 | |
| Oropharynx | Aetiologic fraction | 0·70 | 0·92 | 0·97 | 0·84 |
| | Case fraction | 0·14 | 0·40 | 0·38 | |
| Hypopharynx | Aetiologic fraction | 0·84 | 0·72 | 0·94 | 0·75 |
| | Case fraction | 0·40 | 0·18 | 0·30 | |
| Larynx | Aetiologic fraction | 0·78 | 0·87 | 0·95 | 0·78 |
| | Case fraction | 0·25 | 0·34 | 0·31 | |
| Oesophagus | Aetiologic fraction | 0·61 | 0·54 | 0·84 | 0·50 |
| | Case fraction | 0·33 | 0·22 | 0·22 | |

could be attributable to smoking and/or chewing is highest for cancer of the oropharynx (0·84) and lowest for cancer of the oesophagus (0·50).

Table IV gives the index of synergy (Rothman 1974) with 90% confidence limits, for all the sites studied, for smoking and chewing, assuming the amount smoked

TABLE IV.—*Indices of Synergy Between Chewing and Smoking*

| Site | Index | 90% Confidence limits |
|---|---|---|
| Oral cavity | 1·3 | 1·0–1·8 |
| Oropharynx | 2·3 | 1·9–2·9 |
| Hypopharynx | 2·0 | 1·2–3·5 |
| Larynx | 1·8 | 1·5–2·3 |
| Oesophagus | 1·9 | 1·3–2·8 |

by non-chewing smokers and chewing smokers is similar (and amount chewed by non-smoking chewers and smoking chewers is similar). The values of the index for cancers of the oral cavity, oropharynx, hypopharynx, larynx and oesophagus (1·3, 2·3, 2·0, 1·8 and 1·9, respectively) show that at each of the above sites, smoking and chewing act synergistically, not independently.

### DISCUSSION

It has already been shown by several workers, that smoking and chewing are high risk factors in oral and pharyngeal cancers. But the study of the overall aetiologic fraction related to the habit of smoking and/or chewing, which are very high in cancers of the oral cavity, oropharynx, larynx and hypopharynx show that these are not mere risk factors, but predominant ones in the aetiology of these cancers. The role of public education in eradicating or reducing the addiction to these habits, for bringing these cancers under control, cannot be overemphasized.

On the other hand, for cancer of the oesophagus, even though smoking and chewing are risk factors, only 50% are accounted for by these habits, showing that other factors are of equal importance in the aetiology of cancer at this site. Interestingly, in one of our earlier studies on cancer profiles in various endogamous groups in Western India (Jayant, Balakrishnan and Sanghvi, 1971), the frequency of these 2 habits of smoking and chewing alone, could not explain the pattern of oesophageal cancers in the endogamous groups. It appears that there is not only a need to study the role of drinking habits and diet for this site (as has been done in other parts of the world) but also host susceptibility.

### REFERENCES

JAYANT, K., BALAKRISHNAN, V. & SANGHVI, L. D. (1971) A Note on the Distribution of Cancer in some Endogamous Groups in Western India. *Br. J. Cancer*, **25**, 611.

JUSSAWALLA, D. J. & DESHPANDE, V. A. (1971) Evaluation of Cancer Risk in Tobacco Chewers and Smokers, an Epidemiologic Assessment. *Cancer, N.Y.*, **28**, 244.

LEVIN, M. L. (1953) The Occurrence of Lung Cancer in Man. *Acta Un. Intern. Cancer*, **9**, 531.

MIETTINEN, OLLI, S. (1973) Risk Indicators for Coronary Heart Disease. *Heart Bull.*, **4**, 64.

MIETTINEN, OLLI S. (1974) Proportion of Disease Caused or Prevented by a Given Exposure, Trait or Intervention. *Am. J. Epidemiol.*, **99**, 325.

NIBLOCK, W. J. (1902) Cancer in India. *Indian med. Gaz.*, **27**, 161.

ORR, I. M. (1933) Oral Cancer in Betel Nut Chewers in Travancore. *Lancet*, ii, 575.

ROTHMAN, K. J. (1974) Synergy and Antagonism in Cause Effect Relationships. *Am. J. Epidemiol.*, **99**, 385.

SANGHVI, L. D., RAO, K. C. M. & KHANOLKAR, V. R. (1955) Smoking and Chewing of Tobacco in Relation to Cancer of the Upper Alimentary Tract. *Br. med. J.* i, 1111.